# Rimonabant, Gastrointestinal Motility and Obesity

**DOI:** 10.2174/157015912803217297

**Published:** 2012-09

**Authors:** Yan Sun, Jiande Chen

**Affiliations:** 1 Veterans Research and Education Foundation, VA Medical Center, Oklahoma City, OK, USA; 2 Internal Medicine Department, TTUHSC, El Paso, TX, USA

**Keywords:** Rimonabant, gastrointestinal motility, obesity, food intake.

## Abstract

**Background:**

Obesity and overweight affect more than half of the US population and are associated with a number of diseases. Rimonabant, a cannabinoid receptor 1 blocker in the endocannabinoid (EC) system, was indicated in Europe for the treatment of obesity and overweight patients with associated risk factors but withdrawn on Jan, 2009 because of side effects. Many studies have reported the effects of rimonabant on gastrointestinal (GI) motility and food intake.

**The aims of this review are:**

1. to review the relationship of EC system with GI motility and food intake;
2. to review the studies of rimonabant on GI motility, food intake and obesity;
3. and to report the tolerance and side effects of rimonabant.

**Methods:**

the literature (Pubmed database) was searched using keywords: rimonabant, obesity and GI motility.

**Results:**

GI motility is related with appetite, food intake and nutrients absorption. The EC system inhibits GI motility, reduces emesis and increases food intake; Rimonabant accelerates gastric emptying and intestinal transition but decreases energy metabolism and food intake. There is rapid onset of tolerance to the prokinetic effect of rimonabant. The main side effects of rimonabant are depression and GI symptoms.

**Conclusions:**

Rimonabant has significant effects on energy metabolism and food intake, probably mediated via its effects on GI motility.

## INTRODUCTION

Obesity is widely recognized as a serious health problem with increasing prevalence across the US and the world, and is one of the leading causes of preventable deaths [1]. The annual number of deaths attributed to obesity is estimated to be as high as 400,000. Nearly 70% of the adult U.S. population is overweight or obese [2]. Researches have shown that medical and other costs related to obesity are rising at an alarming rate. Obesity is a multifactorial condition, resulting from imbalance in energy intake and expenditure. Despite the high prevalence of obesity and the need for effective treatments, weight loss remains a difficult goal to achieve. Only a few anti-obesity medications have been approved by the U.S. FDA and several of them were recently withdrawn from the U.S. market. 

The major categories of approved agents for obesity treatment include appetite suppressants, anorexiants and gastrointestinal fat blockers. Appetite suppressing medications have traditionally targeted three monoamine receptor systems in the hypothalamus: noradrenergic, dopaminergic, and serotonergic [3]. The two most commonly used sympathomimetic amines are phentermine and diethylpropion, both labeled as Schedule IV drugs by the Drug Enforcement Agency (DEA). Since these drugs wereinitially approved for only short-term use at a time when obesity was considered an exogenous disorder, no long-term studies have been conducted. Side effects included insomnia, dry mouth, anxiety, constipation, elevations in blood pressure and heart rate. Sibutramine (Reductil or Meridia), a compound that has dual serotonin and norepinephrine reuptake inhibition (SNRI) and primarily affects satiation, was approved by the FDA in 1997 for weight loss and weight maintenance in conjunction with a reduced-calorie diet. Side effects include headache, dry mouth, insomnia, constipation, and elevations in blood pressure and heart rate. Because of serious, non-fatal cardiovascular events, the European Medicines Agency and FDA recommended discontinuation of sibutramine use in European and US market in 2010. Orlistat (Xenical) was approved by the FDA in 1999 as the first lipase inhibitor for obesity management including weight loss and weight maintenance. A number of gastrointestinal tract adverse effects have been reported in at least 10% of orlistat-treated patients: oily spotting, flatus with discharge, fecal urgency, fatty/oily stool, oily evacuation and increased defecation.

There are also other new agents for obesity treatment. Exenatide (Byetta), a 39-amino acid synthetic GLP-1R agonist with longer biological activity than GLP-1, was approved by the FDA in 2005 as adjunctive therapy to improve glycemic control in patients with type 2 diabetes. It must be injected subcutaneously twice daily and causes severe nausea in some patients, especially when therapy is initiated. Byetta is recommended as a weight loss drug only for patients with Type 2 Diabetes. Pramlintide (Symlin), a synthetic, soluble analog of human amylin, was approved by the FDA in 2005 as an antihyperglycemic agent for patients with diabetes as adjunctive therapy to mealtime insulin. Pramlintide administration has previously been shown to reduce caloric intake and increase satiety in short-term studies [4]. The most common adverse event reported was mild and transient nausea. It must be injected at mealtimes. Lorcaserin (Lorquess) is a 5-HT_2C_ receptor agonist. Most frequently reported side effects are headache, dizziness and nausea. Bupropion SR, a dopamine and norepinephrine reuptake inhibitor, and naltrexone SR, an opioid receptor antagonist (Contrave), are combined to tackle the motivation/reinforcement that food brings (dopamine effect) and the pleasure/palatability of eating (opioid effect). Headache, constipation, dizziness, vomiting, and dry mouth were reported to be more frequent in the active medication groups compared to placebo, whereas the most serious side effects are cognitive impairment and. Chinese traditional medicine and acupuncture have been shown to be more effective than placebo or lifestyle modification in reducing body weight in some studies. They would be more attractive if proven effective because they typically have fewer adverse effects. However, these previous studies were limited by small sample size and low quality of experimental methodologies [5].

Drugs blocking the cannabinoid receptors in the endocannabinoid (EC) system may be a future strategy for appetite suppression and energy metabolism. Rimonabant (SR141716) is the first in a new class of agents that appears to work by selectively blocking the cannabinoid-1 receptors (CB-1) in the endocannabinoid system with a chemistry name of N-piperino-5- (4-chlorophenyl)-1-(2, 4-dichlorophenyl) -4-methylpyrazole-3-carboxamide. Phase III clinical trials showed promising effects in the fight against obesity [6-8]. 

The EC system plays an important role in regulation of energy metabolism, food intake and gastrointestinal (GI) motility. The stimulation of CB1 receptors in the EC system is believed to affect central and peripheral actions on lipid and glucose metabolism in adipose tissue and help to regulate food intake, energy balance and GI motility [9]. A number of studies have shown that rimonabant, an antagonist of the CB-1 receptor, exerts significant effects on GI motility and energy metabolism. The aim of this review was to clarify the relationship of EC system with GI motility and food intake, critically review recent studies investigating the effects of rimonabant on GI motility and obesity, and discuss the tolerance and side effects of rimonabant. 

## GASTROINTESTINAL MOTILITY, FOOD INTAKE AND ENDOCANNABINOID SYSTEM

The GI tract plays fundamental roles in the regulation of appetite and digestion of nutrients. Alterations in GI motility have been observed in obese patients, and these alterations could be important factors to the development of obesity and eating disorders [10]. Gastric motility is a key mediator of hunger, satiation and satiety. Gastric accommodation and gastric emptying play important roles in the regulation of gastric distention and intestinal exposure of nutrients and hence control satiation and satiety [11]. Several studies have sought to find a relationship between body size and gastric emptying. The patient with obesity may have accelerated gastric emptying, which was reported to be associated with overeating and obesity in rats [12]. On the other hand, delayed gastric emptying results in early satiety, nausea, vomiting, reduced food intake and weight loss [13, 14]. However, findings on gastric emptying in obese subjects have been controversial and inconsistent; several other confounding factors make it impossible to unequivocally interpret the effect of gastric emptying on the genesis of obesity [15].

Different mechanisms are involved in gastric emptying of solids and liquids, and these cause different effects on food intake and appetite. Gastric emptying of liquid food is driven mainly by the tone of the gastric fundus. An initial acceleration of gastric liquid emptying may reduce symptoms of fullness arising from the stomach, but may lead to a higher rate of energy delivery into the duodenum, resulting in a higher nutrient load to the proximal small intestine and yielding increased fullness or satiation arising from the duodenum. Emptying of solid food requires initial grinding or trituration through antral contractions. Gastric emptying of solids is in a two-phase process, with an initial lag or retention period followed by a linear emptying phase. Persistent delayed gastric solid emptying prolongs the presence of food within the stomach and has been associated with reduced food intake [14]. Relaxation of the proximal stomach, a decrease in antral and duodenal contractility, and an increase in tonic and phasic pyloric pressures lead to delayed gastric emptying [16]. Distension of the proximal stomach with a water-filled balloon in humans is associated with a reduction in energy intake [16]. 

Small bowel transit plays an important role in nutrients absorption that is related to the development of obesity. The degree of absorption of nutrients from the human small intestine is thought to be related to the efficiency of the digestive and epithelial transport mechanisms and the area of the intestine mucosa presented to the luminal contents. Acceleration in intestinal transit may reduce nutrient absorption, enhance satiation due to early triggering of ileal brake, and lead to a weight loss, whereas a delay in intestinal transit may increase absorption and result in weight gain [10]. Undigested nutrients can reach the ileum under normal physiological situations, resulting in release of glucagon-like peptide 1 (GLP-1) and polypeptide YY (PYY), a process called ileal brake [17]. The ileal brake activation reduces food intake and increases satiety levels, which appears to be maintained over the postprandial period. The interaction of fat with receptors in the small intestine results in inhibition of gastric emptying, which serves to prolong gastric distension and regulate the rate at which nutrients enter the small intestine [10]. This negative feedback effect is positively proportional to the length of the small intestine exposed [10].

The EC system includes cannabinoid receptors 1 and 2 (CB1 and CB2), endogenous ligands that activate them and protein involved in endocannabinoid biosynthesis and catabolism [18-20]. The latter occurs through cellular reuptake, which might be facilitated by a putative membrane transporter and enzymatic degradation by hydrolytic enzyme including fatty acid amide hydrolase (FAAH). The EC system regulates energy balance and food intake both in the brain and in the periphery [21-24] through receptor-mediated mechanisms [25]. Cannabinoid receptors, first identified in the brain [26], expressed in the adipocyte, the skeletal muscle, the liver, the pancreas and the enteric nervous system [27]. CB1 receptors are involved in lipid and glucose metabolism, determine appetite, and inhibit gastrointestinal motility [28-30], mediated through activation of the CB1 receptors. The effects of CB1 receptor activation in the GI tract have been previously reviewed [31-37]. CB1 receptor agonists, mostly *via *enteric, vagal, brainstem and spinal nerves, delay gastric emptying and small bowel transit [38], reduce diarrhea, pain and hyperalgesia [28, 29, 39], decrease emesis [40] and gastric acid secretion, as well as promote eating. 

As stated above, delayed gastric emptying is often linked to the sensation of nausea so it would seem paradoxical for an endogenous ligand to affect the stomach in this way while aiming at reducing emesis. The explanation for this apparent contradiction lies in different doses used and different mechanisms and effects of CB1 receptor agonist on liquid and solid gastric emptying. Conversely, selective CB1 receptor antagonists can reduce food intake and body weight, and do so preferentially in obese animals [24]. Such findings have led to the development of selective CB1 receptor antagonists for the treatment of obesity. One such antagonist is rimonabant, which, when combined with a hypocaloric diet over 1–2 years, can promote a clinically significant decrease in body weight and waist circumference, and improvement in cardiovascular risk factors [7, 8, 41].

Other components of the EC system are also involved in the regulation of GI motlity. Bashashati *et al.,* have recently reported that inhibiting FAAH normalizes various parameters of GI dysmotility, such as upper GI transit and fecal output [42]. Inhibitors of endocannabinoid inactivation such as FAAH or MGL inhibitors inhibit GI motility, an effect reduced or abolished by selective CB1 receptor antagonist or in CB1-receptor-deficient mice [43, 44]. To date, there is less evidence that CB2 receptor is involved in the control of normal GI motility. In pathophysiological states, it has been reported that both CB1 and CB2 receptor activation may reduce hypermotility associated with gut inflammation and/or immune activation in rodents. Whether this is true in humans remains to be determined [45, 46]. 

## RIMONABANT, GUT MOTILITY AND OBESITY

### Rimonabant and Gut Motility

As mentioned above, GI motility is closely related to food intake and weight change. Numerous studies have investigated the effects of rimonabant on GI motility. These studies help us understand the mechanisms of the rimonabant actions on obesity. 

#### In Vivo Studies

One study found that administration of rimonabant increased gastric liquid emptying in mice fed a high-fat diet [44], counteracting the cannabinoid agonist effect of delaying gastric emptying [47]. Another study reported that oral or intracerebroventricular (i.c.v.) rimonabant prevented gastric emptying and intestinal transit delay caused by i.c.v. but not intraperitoneal injection (i.p.) of CB1 receptor agonist [48]. Rimonabant was reported to increase small intestinal transit in a dose-dependent manner in mice [28], promote fecal output and increase intestinal fluid volume in rats and gastrointestinal transit in mice [29]. Again in mice, intestinal motility (measured using a fluorescent marker) was reduced by FAAH inhibition *via *a mechanism prevented by rimonabant [43], which was interpreted as a reflection of the inverse agonistic activity of rimonabant at the cannabinoid CB1 receptor that normally inhibits peristalsis. However, no studies have investigated the effect of rimonabant on gastric emptying of solids. In humans, Ameloot *et al.,* reported that rimonabant inhibited the meal-induced gastric accommodation reflex, but did not alter gastric compliance and sensitivity to distension, or meal related symptoms [49].

#### In Vitro Studies

CB1 receptors are located within the myenteric plexus and their activation can reduce excitatory cholinergic neurotransmission in the intestine of various species including humans [50, 51], leading to reduced peristalsis, reduced GI motility and transit *in vivo* [45]. Rimonabant has been reported to increase electrically evoked, cholinergically mediated contractions of the isolated ileum of the guinea pig [52]. Other studies also showed that rimonabant, *in vitro* preparations, enhanced electrically evoked acetylcholine release from myenteric nerves [53] and electrically evoked contractions of myenteric plexus longitudinal muscle obtained from guinea pigs [54, 55]. This excitatory activity is consistent with the ability of rimonabant to increase tonic and phasic activity in isolated mouse colon [56] and increase intestinal motility and defecation in rodents [29, 57]. Prejunctional CB1 receptors were reported to produce inhibition of non-adrenergic non-cholinergic contractile responses in mouse colonic preparations, which was antagonized by rimonabant [58]. Paralytic ileus induced by i.p. administration of acetic acid was also alleviated by rimonabant, suggesting that rimonabant represents a new drug to treat intestinal hypomotility disorders [46]. Two recent studies indicated that in the rat-isolated and guinea-pig isolated myenteric plexus-longitudinal muscle preparation, electrical field stimulation with single and trains of pulses evoked the neurogenic ACh-mediated twitch and rebound contractions, respectively. Rimonabant augmented the twitched contractions, which might be through antagonism of an endocannabinoid tone or inverse agonism [59, 60]. 

### Rimonabant, Food Intake and Obesity

#### Animal Studies

A number of preclinical studies have investigated the effect of rimonabant on food intake in rodent models. One of the earliest studies, using rimonabant in Wistar and Sprague–Dawley mice and rats, suggested that inhibition of an endogenous cannabinoid system might alter the appetite value of highly palatable foods, such as sucrose, in both liquid and oral forms [61]. In another rodent study, rimonabant was found to produce a dose-dependent decrease in food intake without disrupting other normal behaviors [62]. The acute administration of rimonabant has been reported to stimulate the passage of a non-absorbable meal through the small intestine [57, 63, 64] and defecation [57] in mice. In diet-induced obese mouse model [65], a 5-week treatment of rimonabant resulted in a 48% reduction in food intake and a 20% reduction in body weight and facilitated the improvement in insulin resistance. In summary, several animal studies have demonstrated significant weight loss associated with rimonabant. These rodent experiments have been instrumental in laying the initial groundwork pointing to further investigation into the effects of rimonabant on obesity in humans. Most importantly, no significant toxic effects associated with rimonabant were reported in rodents. Further researches showed that the inhibitory effects of rimonabant on food intake and body weight were associated with alteration of leptin expression in the central nerve system [66]. 

#### Clinical Trials

A randomized, double-blind, placebo-controlled crossover study assessed the effect of 7-day oral administration of rimonabant on hunger, satiety, food consumption, and body weight in overweight or obese humans [67]. The results showed reduced hunger and caloric intake with all types of food and reduced body weight with one-week treatment of rimonabant. In a double-blind, placebo-controlled study of 167 patients with various oral doses of rimonabant (5, 10, or 20 mg/day), a dose-dependent reduction in body weight was noted with a weight loss of 4 kg at the highest tested dose at 20mg/day [68].

In the RIO (Rimonabant in Obesity) trail, rimonabant was evaluated in more than 6,600 overweight/obese patients with or without comorbidities with a follow-up of 1 to 2 years [6-8]. Entry criteria included body mass index (calculated as weight in kilogram divided by the square of height in meter) of 30 or greater (obese) or body mass index of higher than 27 (overweight) with treated or untreated dyslipidemia or hypertension. As compared with placebo, rimonabant at a dose of 20 mg was associated with a significant (P<0.001) weight loss (-6.7±0.5 kg with the repeated-measures method and -5.4±0.4 kg with the last-observation-carried-forward analyses). One of the most promising results was in the area of metabolic syndrome: in patients with metabolic syndrome, Rimonabant 20mmg reduced waist circumference (-5.8±0.5 cm with the repeated-measures method -4.7±0.5cm with the last-observation-carried-forward analyses), increased the high density cholesterol level (10.0±1.6% with the repeated-measures method and 8.1±1.5% with the last-observation-carried-forward analyses) and reduced triglycerides (-13.0±3.5 % with the repeated-measures method 12.4±3.2% with the last-observation-carried-forward analyses). Rimonabant at a dose of 20 mg also resulted in an increase in plasma adiponectin level (57.7% with the repeated-measures method and 46.2% with the last-observation-carried-forward analyses) that was partly independent of weight loss.In these clinical studies, a weight regain was noted in the patients who stopped treatment in the second year of the trials, suggesting that the patient may have to take this medication indefinitely to achieve chronic weight loss. In RIO-Diabetes studies [41, 69] rimonabant at a dose of 20 mg/day, in combination with diet and exercise, was found to produce a clinically meaningful reduction in body weight and improve HbA1c and a number of cardiovascular and metabolic risk factors in overweight or obese patients with type 2 diabetes who were inadequately controlled by metformin or sulphonylureas. In a recent small sample size clinical study in patients with schizophrenia, rimonabant did not result in a significant weight loss or improvement in metabolism, but showed a great reduction in brief psychiatry rating scale scores [70]. 

### Tolerance and Adverse Effects of Rimonabant

In a non-obese rodent study with repeated administration of rimonabant [63], tolerance to the anorectic effect of rimonabant was reported to develop over time. However, weight loss persisted well beyond the drug’s effect on food intake. In a study investigating the effects of repeated administration of rimonabant on gastrointestinal propulsion in mice, the acute administration of rimonabant produced a marked stimulation of small intestinal peristalsis [71]. However, tolerance to this effect rapidly developed after repeated treatments and the stimulant effect of rimonabant on the transit of the non-absorbable marker through the small intestine vanished on the third day of treatment [71]. Clarification of the mechanism of the rapid onset of tolerance to the prokinetic effect of rimonabant might help to understand the physiological role of the cannabinoid CB1 receptor in the control of intestinal motility and, more generally, the mechanisms involved in tolerance to cannabinoid agents.

The prokinetic effect of CB1 receptor antagonists in animals is consistent with data from clinical trials that highlighted diarrhea as one of the initial adverse events associated with rimonabant. Von *et al.,* reported that at 1 year, adverse events more frequently related to rimonabant were gastrointestinal, neurological, and psychiatric in nature, and serious adverse events were infrequent and almost equivalent to placebo [72]. Due to the central action of the CB1 receptor agonists, adverse events of severe depression and suicidal thoughts were frequently reported with the use of rimonabant [73-75]. Consequently, on June 15, 2007, the U.S. FDA and on January 16, 2009, the EMEA (European Medicine Agency) had voted not to recommend the drug's approval because of concerns over suicidality, depression and other side effects. One approach to avoid these side effects is to use low doses of rimonabant given over a long period of time, which might improve metabolic risk factors with no evidence of adverse events [72]. An alternative approach is to develop CB1 antagonists that do not cross the blood–brain barrier [76]. However, no clinical data is yet available on these compounds. Furthermore, due to the strong influence of central endocannabinoid-mediated mechanisms on accumulation of peripheral white adipose tissue, they might not be as effective at reducing abdominal obesity as the currently available CB1 antagonists.

## CONCLUSIONS

**Fig. (1):**
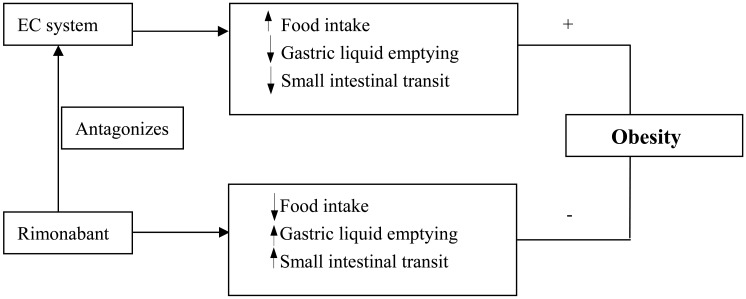
Relationship among EC system, Rimonabant, GI motility and Obesity.

This paper reviewed the effects of the EC system and rimonabant on GI motility, food intake and obesity (Fig. **1**). In brief, in preclinical studies, rimonabant was shown to increase gastric lipid emptying and intestinal transit, and decreases food intake and appetite. In clinical studies, rimonabant was noted to be a promising medicine for obesity treatment due to its inhibitory effect on body weight mediated *via *its effects on GI motility, food intake and energy metabolism. However, the medication has serious side effects on depression and suicide. Future works should be devoted to developing CB1 antagonists that do not cross the blood-brain barrier or improving the chronic use of low dose rimonabant.
